# Point‐of‐care testing for influenza in a university emergency department: A prospective study

**DOI:** 10.1111/irv.12857

**Published:** 2021-04-04

**Authors:** Benjamin Perlitz, Anna Slagman, Jennifer Hitzek, Dorothee Riedlinger, Martin Möckel

**Affiliations:** ^1^ Emergency and Acute Medicine Campus Virchow Klinikum and Campus Charité Mitte Charité Universitätsmedizin Berlin Berlin Germany

**Keywords:** emergency department, influenza, point‐of‐care testing, polymerase chain reaction, staff protection

## Abstract

**Background:**

Seasonal influenza is a burden for emergency departments (ED). The aim of this study was to investigate whether point‐of‐care (POC) PCR testing can be used to reduce staff sick days and improve diagnostic and therapeutic procedures.

**Objectives:**

The aim of this study was to investigate whether point‐of‐care (POC) PCR testing can be used to reduce staff sick days and improve diagnostic and therapeutic procedures.

**Methods:**

Using a cross‐over design, the cobas® Liat® Influenza A/B POC PCR test (Liat) was compared with standard clinical practice during the 2019/2020 influenza season. All adult patients (aged ≥ 18 years) with fever (≥38°C) and respiratory symptoms were included. Primary end points were the prevalence of influenza infections in the ED and staff sick days. Secondary end points were frequency of antiviral and antibacterial therapy, time between admission and test result or treatment initiation, patient disposition, ED length of stay (LOS), and for inpatients mortality and LOS. Nurses were interviewed about handling and integration of POC testing. The occurrence of SARS‐CoV‐2 infections coincided with the second half of the study.

**Results:**

A total of 828 patients were enrolled in the study. All 375 patients of the intervention group were tested with Liat, and 103 patients of them (27.6%) tested positive. During the intervention period, staff sick days were reduced by 34.4% (*P* = .023). Significantly, more patients in the intervention group received antiviral therapy with neuraminidase inhibitors (7.2% vs 3.8%, *P* = .028) and tested patients received antibiotics more frequently (40.0% vs 31.6%, *P* = .033). Patients with POC test were transferred to external hospitals significantly more often (5.6% vs 1.3%, *P* = .01).

**Conclusion:**

We conclude that POC testing for influenza is useful in the ED, especially if it is heavily frequented by patients with respiratory symptoms.

## 1 INTRODUCTION

Influenza virus infections are caused by RNA orthomyxoviruses and occur in seasonal epidemics with onset in the winter months and a strong increase in infection numbers after the turn of the year.^1^, ^2^, ^3^ During the 2018/2019 epidemic in Germany, an estimated 5%‐20% of the population was infected, resulting in an estimated 3.8 million influenza‐associated physician consultations, 40 000 hospitalizations and 5000‐25 000 deaths.^1^, ^3^ Thus, influenza represents a resource‐intensive burden for the healthcare system, the associated costs approximate 145 million euros.^4^, ^5^ In the current COVID‐19 pandemic, public measures like social distancing and wearing face masks may also influence the incidence of influenza, at least in upcoming seasons when these respiratory viruses may be co‐circulating.^2^

Typical influenza symptoms are fever, cough, sore throat, rhinitis, muscle or limb pain, headache, and fatigue. However, only one‐third of patients present with these symptoms.^1^, ^3^, ^6^, ^7^ The course of the disease varies from mild respiratory symptoms to severe and lethal pneumonia. As the symptoms are not specific, it is difficult to clinically distinguish influenza infections from other respiratory tract infections. Testing is necessary to confirm the diagnosis. Patients are infectious for 4‐5 days from the onset of symptoms and transmit the virus mainly by droplet infection and through aerosols. Rapid isolation of suspected cases is, therefore, necessary to protect other patients and medical staff.^1^, ^8^ Elderly patients, pregnant women, and patients with comorbidities (chronic heart or lung disease, metabolic diseases, immunodeficiencies, neurological or neuromuscular diseases, and obesity) are at higher risk for a severe course of the disease, so that their protection is particularly important.^1^ Neuraminidase inhibitors (NAI) are a class of antivirals used to treat influenza when there is a risk of severe complications such as pneumonia, bacterial superinfections, or damage to other organs.^1^, ^9^, ^10^, ^11^ Antiviral therapy should at best be initiated within 48 hours, but no later than 5 days after the onset of symptoms and is only partially effective.^1^, ^10^, ^12^, ^13^, ^14^

Diagnostic gold standard for influenza is a polymerase chain reaction (PCR) test, which is usually performed in a central laboratory.^1^, ^15^ The turnaround time (TAT) depends on several factors including transport, time of the day, and speed of communication of results. Especially after hours and at weekends, the TAT often exceeds 24 hours with centralized analysis. For the emergency department (ED) setting, a long TAT is associated with a prolonged stay of potentially infectious patients and thus an increased risk of infection for other patients and staff, as well as a possibly delayed start of therapy. According to current studies, a POC test performing a RT‐PCR is a promising method with high sensitivity and specificity to enable a faster availability of test results directly in the ED.^16^, ^17^, ^18^, ^19^ Previous studies mainly focused on TAT and have shown strong effects on the length of stay (LOS).^20^, ^21^, ^22^, ^23^ The effects on frequency of antibiotic or antiviral therapy with NAI varied from study to study.^13^, ^19^, ^20^, ^21^ However, the effects and the patient population depend on the role of the ED in the respective healthcare system and the established test procedure, a direct comparison with other countries is not easily possible. This study investigates the effects of an influenza point‐of‐care (POC) PCR test at a tertiary care facility in Germany, for the first time. Primary end points were the prevalence of influenza infections among ED patients presenting with respiratory symptoms and the duration of sick days of ED staff. Secondary end points studied were the frequency of antiviral and antibacterial therapy, as well as the time from patient admission to test results and initiation of therapy, the disposition of patients, and the LOS in the ED. In addition, mortality, LOS in‐hospital, and intensive care unit stays (ICU) of inpatients were investigated.

## 2 METHODS

### 2.1 Study design

In a cross‐over design, the Liat POC test was compared with the established clinical practice of selective, clinically driven central laboratory influenza testing. For this purpose, POC testing was implemented into clinical routine in two ED sites: From 16/12/2019 to 09/02/2020, Liat testing was performed in the ED of Charité Virchow‐Klinikum (CVK), while the control group was recruited at the ED of Charité Campus‐Mitte (CCM). After the eight‐week intervention at CVK, the Liat POC test was used from 10/02/2020 to 25/04/2020 in the ED of CCM and the control group was recruited at CVK.

### 2.2 Participants

In the study, all adult patients (aged ≥ 18 years) were included, in whom a body temperature ≥38°C was measured in the ED or reported within 24 hours prior to ED consultation. Additionally, at least one of the following symptoms had to be present: cough, rhinitis, hoarseness/sore throat, fatigue, headache, muscle pain, aching limbs, or chills. Data of all matching patients were collected in an electronic case report form. Central elements of data collection were, besides a thorough patient characterization, data on the stay at the ED and, in the case of admitted patients, on inpatient therapy.

### 2.3 Influenza‐testing

Roche cobas® Liat® System is a real‐time PCR (RT‐PCR) analyzer that provides a differentiated result for Influenza A and Influenza B within 20 minutes.^19^ Sample material is an oro‐nasopharyngeal swab (BD universal viral transport, 3mL, Flock Flex Mini), which was taken by the nursing staff. The POC PCR device was placed on site in the ED and was operated by the nurses. As part of standard clinical practice, patient samples, from the control group for whom an influenza test was ordered, were tested in the central laboratory. In the central laboratory, the Cepheid Xpert® Xpress Flu/RSV Kit was used to performing a RT‐PCR with a TAT of 20 minutes for positive and 30 minutes for negative results.^15^

In order to determine how well the POC PCR device was implementable into clinical routine, nursing staff of one site was interviewed about the device by means of a questionnaire. They were asked about their satisfaction with sample handling, the integration into clinical routine, the display of results, and the usability of the results.

### 2.4 Outcomes

ED staff sick days were recorded on an anonymized aggregated weekly basis and compared between POC intervention and control periods.

### 2.5 Statistical methods

Data analysis was performed using ibm spss version 27 for Microsoft Windows. The distribution of quantitative data was checked and, due to a lack of symmetry, median and interquartile ranges (IQR) were compared. Due to the unfulfilled normal distribution assumption, statistical significance for quantitative characteristics was calculated using the Mann‐Whitney *U* test. For categorical variables, absolute and relative frequencies were compared using Chi‐Square test. A *P*‐value of <.05 was considered statistically significant.

### 2.6 Ethics

The Charité ethics committee had no reservations about the conduct of the study and approved it (EA2/204/19). The study was registered in the German Clinical Trials Registry (DRKS00019207).

## 3 RESULTS

### 3.1 Description

**FIGURE 1:**
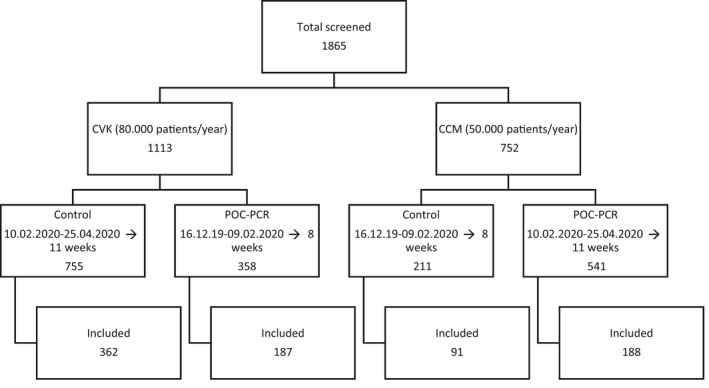
Patient recruitment. Patients were screened for fitting the criteria at two Charité sites (CVK, CCM). All included patients had to be over 18 years of age and presented with fever (≥38°C) and respiratory symptoms

In total, 1865 patients were screened (CVK: 1113, CCM: 752), of which 828 (CVK: 549, CCM: 279) fit the inclusion criteria (Figure 1). All 375 patients of the POC intervention group were tested with Liat. Four hundred and fifty‐three patients were in the control group, of which 244 (53.9%) were tested on a clinical routine basis in the central laboratory. Two hundred and nine patients (46.1%) in the control group did not have clinician‐ordered influenza tests despite fulfilling the inclusion criteria; hence, the control group consists of patients with and without influenza‐testing.

Of 619 patients tested, 185 (29.8%) tested positive for influenza (Influenza A: n = 160, 25.8%; Influenza B: n = 25, 4.0%). In only two cases (0.3%), an influenza infection could not be excluded due to invalid results on the POC PCR device, so that a total of 432 (69.8%) patients were definitively tested negative. The positive rate in the control group was lower than in the intervention group (18.1% vs 27.5%; *P* < .001). Patients who tested positive for influenza were more likely to present with cough (*P* < .001), headache or limb/muscle pain (*P* = .030) or rhinitis (*P* = .029), and less likely with dyspnea (*P* < .001) compared to those who tested negative (Table 1). The time from admission to test result was significantly reduced by 15 minutes in the POC intervention group (52 vs 67 minutes, *P* < .001).

**TABLE 1:** Baseline characteristics

Baseline characteristics	Total	Intervention group	Control group	*P*‐value
Case numbers	828	375	453	
Age	42 (IQR 29‐64)	43 (IQR 29‐64)	42 (IQR 29‐62)	.898
Sex				
Female	399 (48.2%)	174 (46.4%)	225 (49.7%)	.349
Male	429 (51.8%)	201 (53.6%)	228 (50.3%)	
Risk factor: smoking*	158 (18.1%)	86 (22.9%)	72 (15.9%)	.063
Risk factor: alcohol*	71 (8.6%)	27 (7.2%)	44 (9.7%)	.47
Vital parameters				
Blood pressure				
Systolic	128 (IQR 118‐140)	127 (IQR 115‐140)	130 (IQR 119‐141)	.055
Diastolic	78 (IQR 69‐87)	77 (IQR 69‐87)	78 (IQR 69‐86.25)	.557
Heart rate	102 (IQR 89‐115)	102 (IQR 90‐117)	102 (IQR 89‐115)	.596
Body temperature	38.7 (IQR 38.2‐39.1)	38.8 (IQR 38.3‐39.2)	38.6 (IQR 38.1‐39.1)	.001
Respiratory rate	16 (IQR 15‐20)	16 (IQR 15‐20)	16 (IQR 15‐20)	.879
Oxygen saturation	98 (IQR 96‐100)	98 (IQR 95‐100)	98 (IQR 96‐100)	.23
Symptoms				
Fever	828 (100%)	375 (100%)	453 (100%)	
Chills	150 (24.8%)	68 (18.9%)	82 (33.3%)	<.001
Cough	496 (66.0%)	223 (60.9%)	273 (70.9%)	.004
Dry cough	282 (36.5%)	111 (30.5%)	171 (41.8%)	.001
Productive cough	216 (29.3%)	112 (30.8%)	104 (27.9%)	.39
Sore throat/Hoarseness	180 (26.4%)	74 (20.4%)	106 (33.2%)	<.001
Rhinitis	66 (10.3%)	32 (8.9%)	34 (12.3%)	.161
Headache, aching limbs, muscle pain	377 (52.7%)	176 (48.5%)	201 (57.1%)	.021
Dyspnea	186 (25.8%)	96 (26.2%)	90 (25.4%)	.805
Fatigue	345 (51.0%)	144 (39.8%)	201 (64.0%)	<.001
Symptom onset				
0‐48 h	408 (55.7%)	193 (55.9%)	215 (55.4%)	.559
49‐120 h	193 (26.3%)	95 (27.5%)	98 (25.3%)	
>120 h	132 (18%)	57 (16.5%)	75 (19.3%)	
Charlson comorbidity score	1 (IQR 0‐2)	1 (IQR 0‐2)	0 (IQR 0‐2)	.032
Immune suppression	133 (16.1%)	59 (15.7%)	74 (16.3%)	.234
Diabetes mellitus	97 (11.7%)	50 (13.3%)	47 (10.4%)	.009
Organ transplantation	26 (3.1%)	15 (4.0%)	11 (2.4%)	.002
Oncological disease	137 (16.5%)	59 (15.7%)	78 (17.2%)	.343
Cardiovascular diseases	273 (33.0%)	128 (34.1%)	145 (32.0%)	.155
Respiratory diseases	154 (18.6%)	75 (20.0%)	79 (17.4%)	.067
Kidney diseases	95 (11.5%)	53 (14.1%)	42 (9.3%)	.001
Liver diseases	61 (7.4%)	31 (8.3%)	30 (6.6%)	.02
Pregnancy (women only)*	11 (2.8%)	6 (3.4%)	5 (2.2%)	.01
Laboratory values				
pH	7.413 (IQR 7.386‐7.442)	7.413 (IQR 7.386‐7.446)	7.413 (IQR 7.385‐7.439)	.542
Sodium	138 (IQR 135‐140)	138 (IQR 135‐140)	138 (IQR 135‐140)	.806
Potassium	4.0 (IQR 3.7‐4.3)	4.0 (IQR 3.7‐4.3)	3.9 (IQR 3.7‐4.3)	.395
Glucose	118 (IQR 106‐135)	118 (IQR 105‐137)	118 (IQR 106‐132)	.876
Hemoglobin	13.5 (IQR 12.2‐14.8)	13.5 (IQR 12.0‐15.0)	13.6 (IQR 12.3‐14.7)	.56
Lactate	13 (IQR 10‐18)	13 (IQR 9‐18)	13 (IQR 10‐18)	.981
D‐dimers	0.81 (IQR 0.46‐1.31)	0.67 (IQR 0.40‐7.62)	0.85 (IQR 0.56‐1.31)	.563
Leukocytes	9.2 (IQR 6.0‐13.3)	9.3 (IQR 6.3‐13.4)	8.8 (IQR 5.8‐13.2)	.333
Lymphocytes	0.91 (IQR 0.57‐1.39)	0.82 (IQR 0.53‐1.33)	0.95 (IQR 0.60‐1.40)	.44
CRP	43.0 (IQR 15.3‐96.1)	44.4 (IQR 14.5‐92.1)	39.7 (IQR 15.5‐104.0)	.61
LDH	268 (IQR 225‐339)	300 (IQR 227‐348)	260 (IQR 223‐332)	.122
PCT	0.14 (IQR 0.07‐0.47)	0.14 (IQR 0.07‐0.64)	0.13 (IQR 0.06‐0.4)3)	.77
Diagnostics				
Sonography	125 (15.1%)	61 (16.3%)	64 (14.1%)	.392
CT	92 (11.1%)	48 (12.8%)	44 (9.7%)	.16
X‐ray	439 (53.0%)	203 (54.1%)	236 (52.1%)	.559
Influenza testing				
Time interval admission‐test result	58 (IQR 41‐108)	52 (IQR 37‐84)	67 (IQR 48‐155)	<.001
Tested	619 (74.8%)	375 (100%)	244 (53.9%)	<.001
Result				
Negative	432 (69.8%)	270 (72.0%)	162 (66.4%)	.259
Influenza A positive	160 (25.8%	89 (23.7%)	71 (29.1%	
Influenza B positive	25 (4.0%)	14 (3.7%)	11 (4.5%	
Invalid	2 (0.3%)	2 (1.0%)	0 (0%)	

Note

Units: age years; time minutes; blood pressure, pO2, pCO2 mm Hg; heart/respiratory rate /min; oxygen saturation %, sodium, potassium, chloride, calcium, glucose mmol/L; hemoglobin, lactate mg/dL; leukocytes, lymphocytes/mL; D‐dimers, CRP mg/L; LDH U/L; PCT µg/L. Baseline characteristics of the study population distinguished between both study groups. Valid values were used, only for parameters marked with “*” all data were used because the valid data are distorted.

### 3.2 Sick days of ED staff

**FIGURE 2:**
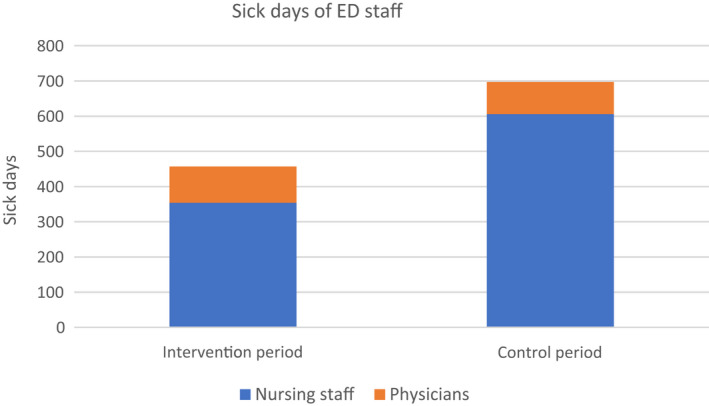
Sick days of emergency department (ED) staff by study period and function. ED staff sick days distinguished between nurses and physicians during both study periods were aggregated and compared with each other

The amount of sick days of ED nurses was significantly reduced in the POC intervention period: In the control period, there was a total of 697 sick days, of which 91 were recorded among physicians and 606 among nursing staff (Figure 2). During the intervention period, there was an overall 34.4% reduction to 457 sick days (*P* = .023); although there was a slight increase in sick days for physicians to 103 (+13.2%, *P* = .506), there was a significant reduction in sick days for nurses to 354 (−41.6%, *P* = .005).

### 3.3 ED therapy

**FIGURE 3:**
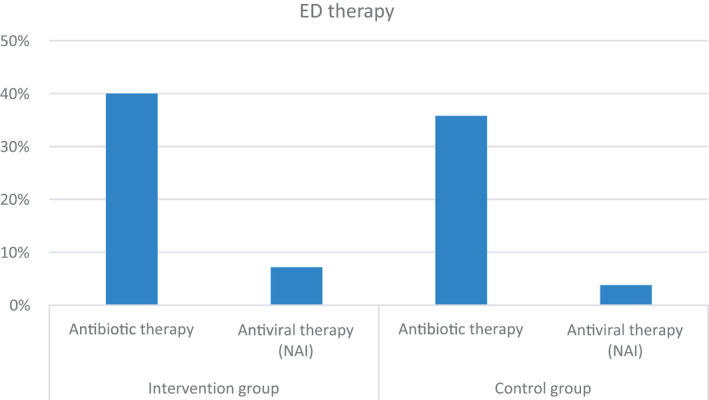
Emergency department therapy. Antibiotic therapy and therapy with antivirals (neuraminidase inhibitors) were compared between both study groups

Three hundred and twelve patients (37.7%) received antibiotics in the ED (Figure 3). The proportion of patients receiving antibiotic treatment is higher in the intervention group as compared to the control group (40.0% vs 35.8%, *P* = .211), especially compared with the tested subgroup in the control group (40.0% vs 31.6%, *P* = .033; Table 2). Differences in antibiotic prescribing are particularly evident in the patients tested negative for influenza (48.5% vs 39.5%, *P* = .069). In addition, if the influenza test was performed in the ED instead of the central laboratory, antibiotic therapy was initiated 49 minutes earlier (218 vs 169 minutes, *P* = .004). 5.3% of the study population was treated with NAI in the ED as influenza‐specific treatment. In the intervention group, proportionately more patients were treated with NAI than in the control group (7.2% vs 3.8%, *P* = .028), but there were no significant differences in treatment when compared within the subgroups where influenza infection was confirmed by a test (26.2% vs 20.7%, *P* = .912). No patient without a test result or with a negative test result was treated with NAI. Regardless of the study group, the longer the symptom onset, the less NAI was administered (0‐48 hours 29.6%, 49‐120 hours 12.8%, >120 hours 12.5%, *P* = .021). In the POC intervention group, NAI therapy was initiated 82 minutes faster (244 vs 162 minutes, *P* = .024).

**TABLE 2:** ED therapy and disposition

ED therapy and disposition	(sub‐) group	Total	Intervention group	Control group	*P*‐value
Antibiotics	All	312 (37.7%)	150 (40.0%)	162 (35.8%)	.211
	Tested	227 (36.7%)	150 (40.0%)	77 (31.6%)	.033
	Positive	32 (17.3%)	19 (18.4%)	13 (15.9%)	.644
	Negative	195 (45.1%)	131 (48.5%)	64 (39.5%)	.069
Time interval admission‐antibiotics (min)	All	171 (IQR 107‐260)	169 (IQR 96‐255)	175 (IQR 122‐287)	.09
	Tested	176 (IQR 108‐267)	169 (IQR 96‐255)	218 (IQR 139‐302)	.004
Antiviral therapy (NAI)	All	44 (5.3%)	27 (7.2%)	17 (3.8%)	.028
	Tested	44 (7.1%)	27 (7.2%)	17 (7.0%)	.912
	Positive	44 (23.8%)	27 (26.2%)	17 (20.7%)	.386
	Negative	0	0	0	
Antiviral therapy (NAI) by symptom onset					
0‐48 h	All	32 (29.6%)	18 (29.5%)	14 (29.8%)	.014
49‐120 h		6 (12.8%)	4 (18.2%)	2 (8.0%)	
>120 h		2 (12.5%)	2 (20.0%)	0 (0.0%)	
Time interval admissions—antiviral therapy (NAI) (min)	All	211 (IQR 145‐268)	162 (IQR 111‐258)	243 (IQR 197‐293)	.023
	Tested	209 (IQR 143‐271)	162 (IQR 111‐258)	244 (IQR 190‐298)	.024
Disposition					
Discharged home	All	480 (58.0%)	220 (58.7%)	260 (57.4%)	.010
	Tested	363 (58.6%)	220 (58.7%	143 (58.6%)	.051
	Positive	138 (74.6%)	82 (79.6%)	56 (68.3%)	.005
	Negative	224 (51.9%)	137 (50.7%)	87 (53.7%)	.3
Internal admission	All	321 (38.8%)	134 (35.7%)	187 (41.3%)	.010
	Tested	232 (37.5%)	134 (35.7%)	98 (40.2%)	.051
	Positive	43 (23.2%)	17 (16.5%)	26 (31.7%)	.005
	Negative	188 (43.5%)	116 (43.0%)	72 (44.4%)	.3
External admission	All	27 (3.3%)	21 (5.6%)	6 (1.3%)	.010
	Tested	24 (3.9%)	21 (5.6%)	3 (1.2%)	.051
	Positive	4 (2.2%)	4 (3.9%)	0 (0.0%)	.005
	Negative	20 (4.6%)	17 (6.3%)	3 (1.9%)	.3
LOS (min)	All	251 (IQR 156‐364)	254 (IQR 159‐368)	250 (IQR 149‐363)	.342
	Tested	262 (IQR 176‐385)	254 (IQR 159‐368)	276 (IQR 199‐403)	.09
	Positive	249 (IQR 182‐356)	225 (IQR 138‐338)	264 (IQR 182‐356)	.002
	Negative	273 (IQR 166‐405)	261 (IQR 166‐388)	282 (IQR 165‐417)	.812

Note

ED therapy and disposition is shown for both study groups in general and for the distinguished subgroups named in the second column. The percentages refer to the subgroup named in the second column.

### 3.4 Inpatient admission and ED disposition

**FIGURE 4:**
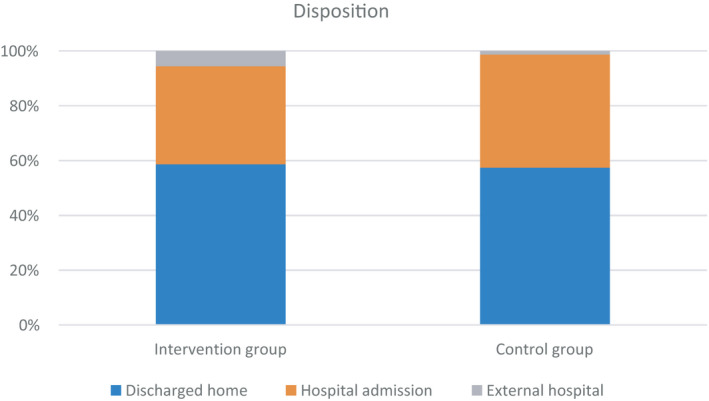
Disposition by study groups. Disposition from the emergency department is shown for both study groups

Three hundred and twelve patients (38.8%) were admitted from the ED to the Charité hospital as inpatients. Further 27 patients (3.3%) were transferred to external hospitals and 480 patients (58%) were discharged home (Figure 4). Although there were no relevant differences between the study groups regarding discharged home and admission to hospital in general, differences in disposition between the groups were observed. Patients of the POC intervention group were more often transferred to external hospitals than patients of the control group (5.6% vs 1.3%, *P* = .010). This difference between both groups could also be observed in patients tested positive (3.9% vs 0.0%, *P* = .005). The LOS in the ED of the intervention group was 39 minutes shorter in influenza‐positive tested patients (264 [IQR 182‐356] vs 225 minutes [IQR 138‐338, *P* = .002]).

### 3.5 In‐Hospital therapy and mortality

Compared with ED treatment, no differences between the study groups were shown in frequency of inpatient antiviral or antibiotic therapy. However, median LOS was 2 days longer in the intervention group (9 vs 7 days, *P* = .026). This difference is even more pronounced when compared to the tested subgroup (9 vs 6 days, *P* = .003). 23.1% of the hospitalized patients were in the ICU, with no significant difference between the study groups. Nevertheless, more patients in the intervention group were ventilated (n = 52, 20.1%, vs 13.4%, *P* = .105) and the mortality of inpatients was slightly higher than in the control group (6.7% vs 4.8%, *P* = .465).

### 3.6 Employee satisfaction survey

The questionnaire was distributed to 40 nurses; 25 of whom replied. Most respondents (60%) had performed more than 25 POC tests. 52% of all respondents said they were "satisfied" with the handling of the samples and 36% said they were "neither satisfied nor dissatisfied" with it. 60% of respondents were at least "satisfied" with the integration of POC testing in clinical routine and 76% said it could be easily integrated into ED care. In addition, 56% said that the POC influenza results influenced their patient management. The main reason for dissatisfaction was that results could neither be printed nor be fed directly into the hospital or laboratory information system (HIS/LIS). Another point of criticism was that the sample handling was considered cumbersome and sometimes even unhygienic. A total of 92% of those surveyed stated that the topic of infectious diseases should gain more attention overall.

## 4 DISCUSSION

These results show that influenza POC testing in the ED is a useful diagnostic tool, especially during an influenza wave.

Short test duration and direct availability of POC results made it possible to detect more influenza infections and initiate therapy significantly earlier although the time to obtain a test result for ED patients by central laboratory testing in this study was lower than in other studies.^20^, ^21^ Since the sensitivity (98.8%) and specificity (98.5%) of POC PCR using Liat is very high and invalid results are rare, a reliable result that influenced the adoption of isolation measures and initiation of therapy was available very quickly.^16^, ^17^, ^18^, ^19^ Feedback from nursing staff shows that it could be implemented into clinical routine easily.

Of particular importance is that the unlimited availability of a POC test resulted in more patients tested for influenza in the ED, and thus, infections not initially suspected have been detected. It can be assumed that due to the existing symptoms (49.1% cough, 49.1% headache, muscle pain, or aching limbs), many patients in the untested part of the control group should have been isolated, as these symptoms were significantly more frequent in influenza‐positive patients. Patients with an undetected influenza infection represent a source of infection for others, especially for the staff. The reduction in sick days during the intervention phase may be explained by the shorter LOS of patients with detected influenza in the ED. The reduced LOS in the ED was also observed in other studies, but there are currently no other studies on staff sick days reduction.^20^, ^21^, ^24^, ^25^ Furthermore, the POC test result was used for early identification of influenza‐positive patients during the early phase of the COVID‐19 pandemic and a combination of influenza POC testing with SARS‐CoV‐2 PCR in the ED could further improve infection control early in ED processes. The frequency of antibiotic therapy was not reduced in patients who received a POC test, especially not in those who tested positive, although this was shown in other studies.^21^, ^23^ The reasons for this finding could be that in these studies the TAT of central laboratory testing was significantly higher than in this study and that patients of the intervention group had more comorbidities than patients of the control group, and therefore, antibiotics were more likely to be prescribed to prevent coinfections. In standard clinical practice, the influenza test result is entered into the HIS by the central laboratory, but the medical staff is not informed of an existing result. It is, therefore, obvious that the attending physician only learned of the result during a later review. This “time to brain” or time to “actionable result” is additionally shortened by POC testing.^26^ Furthermore, the Liat can now be implemented in the LIS and HIS and it is possible to connect a printer, which has improved key points of criticism. Other studies showed that the POC test influences the decision to treat with NAI.^22^, ^27^ This could not be confirmed, as the proportion of influenza‐positive patients treated with NAI was comparable between both groups, though significantly more patients overall were treated with NAI in the POC intervention group than in the control group. It can be assumed that therapy was not started until a test result was available, since no patient was empirically treated with NAI. Nevertheless, due to the easy availability of a POC test in the ED, more symptomatic patients were tested, and thus, more patients infected with influenza were identified. In particular, faster treatment with NAI led to lower mortality and LOS in several studies.^13^, ^21^, ^22^, ^23^, ^27^ This could not be confirmed. However, this is probably not related to faster testing, but more likely related to the disposition of patients and characteristics of the study groups. Although the number of inpatients was comparable between both groups, significantly more patients in the intervention group were transferred to external facilities. It can be assumed that mainly severely ill patients were treated at the study sites and that the intervention group had more comorbidities, which is a risk factor for severe disease progression.^1^ It is, therefore, likely that mostly less severe ill patients were transferred. This finding is particularly relevant during severe waves of influenza, but also in view of the COVID‐19 pandemic. It seems that it is well possible to optimize in‐hospital flow by transferring infectious patients with mild courses to peripheral hospitals as soon as the infection has been confirmed by a test. Available POC testing could make it possible to reserve capacities of maximum care providers for severely ill patients. In addition, the same applies to confirmed negative patients who could also be transferred more easily.

### 4.1 Strengths and limitations

Although this is a prospective study, a large part of the patient data was extracted retrospectively from the HIS. The advantage was a large, unselected study population because informed consent was not required but resulted in some missing data. Despite a large study population, only a small portion of patients were treated with NAI. Furthermore, it should be noted that it was not possible to follow up the clinical course of the externally transferred patients. Nevertheless, it seems plausible that patients with severe courses were further treated at Charité. For reasons of data protection law, it was not possible to directly correlate sick days of the staff with identified influenza infections and the difference in sick days may be attributed to a high frequency of sick days during a short time of one control period. Nevertheless, this is the only study to date that has investigated the association between influenza POC testing in the ED and staff sick days. It should also be noted that during the study period, the COVID‐19 pandemic reached Germany, and thus, general hygiene measures such as the permanent wearing of masks, were also implemented in the ED since the end of March. Since mid‐February, SARS‐CoV‐2 and influenza testing had been linked, so more patients were recruited in the second part of the study period, but this affects both control and intervention groups equally. In addition, due to the data structure, it cannot be said with certainty that these are unconnected samples because patients may have presented more than once during the study period.

## 5 CONCLUSION

POC influenza PCR testing significantly reduced the sick days of staff in the ED. The POC testing was easily integrated in routine procedures and run by ED nurses. The indication for treatment with NAI (in positive cases) and antibiotics (in negative cases) was more precise. The transfer to external hospitals was enhanced by the early availability of the influenza status. We conclude that POC testing for influenza is useful in the ED, especially if it is heavily frequented by patients with respiratory symptoms.

## CONFLICT OF INTEREST

Mr Perlitz, Ms Riedlinger, and Ms Hitzek have no conflicts of interest. Prof. Slagman reports grants from Deutsche Forschungsgemeinschaft, grants from Bundesministerium für Bildung und Forschung, grants from Zentralinstitut für die Kassenärztliche Versorgung and grants from Thermo Fisher Scientific outside the submitted work, and grants from Roche Diagnostics related to the submitted work. Prof. Möckel declares research funding from Roche Diagnostics and speaker and advisory board fees from Roche Diagnostics.

## AUTHOR CONTRIBUTION

**Benjamin Perlitz:** Data curation (lead); Formal analysis (lead); Investigation (equal); Methodology (equal); Validation (lead); Visualization (lead); Writing – original draft (lead); Writing – review and editing (lead). **Anna Slagman:** Conceptualization (equal); Formal analysis (equal); Funding acquisition (equal); Investigation (equal); Methodology (equal); Project administration (equal); Supervision (equal); Writing – review and editing (equal). **Jennifer Hitzek:** Data curation (equal); Methodology (equal); Software (lead); Writing – review and editing (supporting). **Dorothee Riedlinger:** Conceptualization (supporting); Methodology (equal); Writing – review and editing (supporting). **Martin Möckel:** Conceptualization (equal); Funding acquisition (lead); Methodology (equal); Project administration (lead); Supervision (equal); Validation (equal); Writing – review and editing (supporting).

### PEER REVIEW

The peer review history for this article is available at https://publons.com/publon/10.1111/irv.12857.

## Data Availability

Research data are not shared.
